# Anti-radiation effect of MRN-100: a hydro-ferrate fluid, *in vivo*

**DOI:** 10.1093/jrr/rrad095

**Published:** 2024-01-21

**Authors:** Mamdooh Ghoneum, Nariman K Badr El-Din, Mai Alaa El-Dein

**Affiliations:** Department of Surgery, Charles Drew University of Medicine and Science, 1621 East 120th Street, Los Angeles, California 90059, USA; Department of Surgery, University of California Los Angeles, 405 Hilgard Ave, Los Angeles, CA 90095, USA; Department of Zoology, Faculty of Science, Mansoura University, Mansoura 35516, Egypt; Department of Zoology, Faculty of Science, Mansoura University, Mansoura 35516, Egypt

**Keywords:** MRN-100, radiation, antioxidant, hematology, histopathology, 8-OHdG, DNA damage

## Abstract

Ionizing radiation (IR) severely harms many organs, especially the hematopoietic tissue, mandating the development of protective nutraceuticals. MRN-100, a hydro-ferrate fluid, has been shown to protect *γ*-radiated fish against hematopoietic tissue damage and lethality. The current study aimed to examine MRN-100’s protective effect against irradiated mice and explore the mechanisms underlying its effect. Mice received a single acute, sub-lethal, 5 Gy, whole body dose of X-ray IR. MRN-100 treatment was administered daily for 2-weeks pre-irradiation until 1-week post-irradiation. Spleen and blood were analysed for oxidative stress, hematological, histological and biochemical parameters. Radiation exposure markedly decreased complete blood count (CBC) parameters including hemoglobin, hematocrit, red blood cells, platelets, white blood cells and lymphocytes, and significantly increased neutrophils. In contrast, MRN-100 supplementation to irradiated mice ameliorated all CBC parameters and protected against DNA damage in both splenic cells and serum. It also had an antioxidant effect, increasing the levels of glutathione, superoxide dismutase, catalase and total antioxidant capacity, which were otherwise decreased by irradiation. MRN-100 intake reduced the oxidative stress biomarker levels of nitric oxide, protein carbonyl, malondialdehyde, reactive oxygen species and 8-hydroxydeoxyguanosine, a marker specific to DNA damage. Furthermore, MRN-100 enhanced serum iron and reversed the radiation-induced elevations of liver enzymes. Finally, MRN-100 protected splenic tissue from irradiation as observed by histology. We conclude that MRN-100 consumption may protect against oxidative stress generated by radiation exposure, suggesting that it may be employed as an adjuvant treatment to prevent radiation’s severe damage to important organs.

## INTRODUCTION

Our natural environmental background inevitably exposes us to ionizing radiation (IR). Interestingly, the total radiation dose annually received from natural sources is approximately the same as the effective dose received from artificial radiation sources (e.g. linear accelerators, X-ray machines, nuclear reactors) [1]. Exposure to IR can severely damage the body’s organs, tissues and cells, including those of the immune system. This can lead to widespread health concerns for medical staff and patients in healthcare settings as well as for populations exposed to accidental environmental contaminations such as the 1986 Ukrainian Chernobyl disaster or the 2011 radiation release from Fukushima in Japan. Radiation affects organs like the spleen and bone marrow, organs vital for proper functioning of the immune system and critical for determining mortality and morbidity after exposure [2, 3]. Other side-effects of radiation include oxidative stress [4] and oxidative damage to cellular macromolecules [5–7], leading to hematopoietic tissue failure [8, 9].

Radiation damage can also be used for beneficial ends—many cancer types continue to be treated with radiotherapy. However, the effectiveness of radiotherapy is limited by the radioresistance of cancer cells [10, 11]. Killing tumors successfully requires the use of a very high radiation dose. Those high doses in turn cause unwanted complications in normal tissues surrounding the tumors as well as in distant organs. Radiotherapy is severely limited by these harmful consequences [12], and it remains a significant challenge for researchers and radiation oncologists to develop approaches that can effectively utilize radiotherapy’s antitumor abilities while also minimizing the dose of radiation and evading its detrimental consequences [13].

Several synthetic radioprotective compounds have been discovered, but their use is restricted due to prohibitively high cost and toxicity. For example, thiol synthetic compounds such as amifostine (2-(3-aminopropylamino) ethylsulfanylphosphonic acid) decrease mortality but are associated with adverse effects. We are in urgent need of alternatives which are very effective while also being less toxic and less costly. MRN-100, a hydro-ferrate fluid, is an iron-based compound composed of bi- and trivalent ferrates isolated from phytosin, and it has been shown to protect *γ*-radiated fish from hematopoietic tissue damage and lethality [14]. In that study, the irradiated fish survival rate was significantly reduced to 27.7% of the control value, while irradiated fish treated with MRN-100 had a survival rate of 87.2%. MRN-100 furthermore protected various organs’ histopathology. In addition, MRN-100 has been shown to be a powerful antioxidant in mice, as evidenced by its augmentation of the antioxidant defense system, modulation of lipid peroxidation (LPx) and prevention of free radical formation [15, 16]. Furthermore, MRN-100 is well known for its established health benefits such as the promotion of anti-inflammatory responses *in vitro* [17], anti-HIV activity *in vitro* [18] and anti-cancer activity in rats with chemically induced esophageal and gastric cancer [19]. Therefore, we hypothesized that MRN-100 would have a protective effect on hematopoietic tissue after exposure to X-ray irradiation and we carried out the current study to examine this potential effect in mice. A sub-lethal dose of whole-body irradiation was studied to draw more direct comparisons with physicians and patients who regularly use (or are regularly exposed to) sub-lethal dosages of X-ray radiation.

## MATERIALS AND METHODS

### Animals

We used Swiss albino mice (40 males, 2 month old, 22 ± 2 g weight) obtained from Cairo University’s National Cancer Institute (Cairo, Egypt). Mice were housed in our animal research facility with 5 mice/cage in alternating 12-h light/dark cycles, 10% relative humidity and constant temperature (24 ± 2°C). They were accommodated for 1 week before the experiments. Mice were fed standard food pellets and provided water *ad libitum*. Mice protocols maintained compliance with the Guide for the Care and Use of Laboratory Animals at the University of Mansoura, Egypt, and all animal experiments were approved by the University of Mansoura, Egypt, approval number Sci-Z-P-110.

### MRN-100

MRN-100 was prepared as described before [19] with Fe^2+^ and Fe^3+^ ion final concentrations of ~2 × 10^−12^ mol/l. Iron concentration in tap water (in the form of ferric) is typically found to range from 0.00 to 0.03 mg/l (0.0–5.4 × 10^−7^ mol/l). MRN-100 is procured from phytosin, a plant extract found in radish seeds, wheat and rice that contains neutral lipid compounds and iron [20]. After phytosin is dispersed in water, ferric chloride is added, lipid compounds are removed and the remaining iron compound is subjected to fractional determination with respect to bi- and trivalent ferrates for MRN-100 generation. MRN-100 was graciously provided by ACM Co., Ltd, Japan.

### Irradiation

Mice received a single whole-body acute dose of X-ray IR with a sub-lethal 5 Gray (GyX rays) dose with a target object distance of 30 cm and an exposure rate of 250 cGy/min. The linear accelerator machine (LINAC) in the oncology center of Mansoura University, Egypt, was used for performing X-ray irradiation.

### Experimental design

Forty mice were randomly separated into four groups (G1–G4) as follows. Group G1 was the untreated vehicle control group, drinking tap water (received neither radiation nor MRN-100). Group G2 received only MRN-100 everyday *ad libitum* for 3 weeks. Group G3 served as the irradiated control group; mice received whole body radiation (5 GyX rays) and drank tap water *ad libitum*. Group G4 was treated with MRN-100 for 2 weeks prior to irradiation, then exposed to whole body irradiation (5 GyX rays) with MRN-100 treatment continuing everyday *ad libitum*. Body weights (BWs) were tracked for the entire experimental duration. The radiation dose was sub-lethal and all mice survived through Day 7.At Day 7 after irradiation, all mice were euthanized. Spleens were investigated for evidence of oxidative stress and antioxidant status. In addition, different biochemical, histopathological and hematological parameters were measured in blood and spleen tissue.

### Body weight

All mice’s initial BW were measured, followed by measurements at intervals of 1, 2 and 3 weeks from the begining of the experiment. BW differences of various treatment groups were compared against those of the control untreated mice.

### Spleen weight and relative spleen weight changes

Spleen weight was examined at Day 7 post-exposure to irradiation, and relative spleen weight was calculated: (spleen weight/BW) × 100.

### Sample collection

On Day 21 (the experiment’s end), mice were fasted for 16 h, following which diethyl ether was used to anesthetize them. Blood from the abdominal aorta was drawn using vacuum tubes and clotted at room temperature. Serum separation was achieved via centrifugation (15 min at 3000 rpm with a centrifuge radius of 10 cm), stored until assayed and then used for determining liver function enzymes and iron profile.

### Hematology studies

At Day 7 after radiation exposure, heparinized plastic syringes were used to draw blood samples via heart puncture. Blood was rapidly transferred into anticoagulation test tubes for CBC analysis of total platelet count, hematocrit (Hct), hemoglobin (Hb), total red blood cell count (RBCs) and total white blood cell (WBCs) with differential counts.

### Biochemical analysis

Mice spleens were dissected at 7 days after radiation exposure and assessed for various oxidative stress parameters. Spleens were homogenized in ice-cold potassium phosphate buffer solution (0.1 mol/l, pH 7.4) using a Potter-Elvehjem homogenizer to give a 10% W/V homogenate. Spleen tissue homogenates were prepared and used to obtain glutathione (GSH), superoxide dismutase (SOD), catalase (CAT) and total antioxidant capacity (TAC) level estimations. GSH content was determined as previously described by Beutler *et al*. [21], SOD activity as in Minami and Yoshikawa [22] and CAT activity according to Luck [23]. TAC level was measured using Randox total antioxidant status kit (UK) according to Rice-Evans and Miller [24]. LPx product malondialdehyde (MDA) was measured as described by Yoshioka *et al*. [25]. Nitric oxide (NO) and protein carbonyl (PCO) in spleen homogenates were estimated according to Miranda *et al*. [26] and Levine *et al*. [27], respectively. Reactive oxygen species (ROS) was assessed by Rat ROS ELISA Kit (Catalogue Number SL1189Ra, Sun Long Biotech Co., LTD, China). 8-hydroxydeoxyguanosine (8-OHdG), the DNA adduct for oxidative damage, was estimated using Rat 8-OHdG ELISA Kit (Catalog Number CSB-E10526r, Cusabio Technology, LLC, USA).

### Liver function enzymes

The serum levels of alanine aminotransferase (ALT) and aspartate aminotransferase (AST) were detected according to Breuer [28] and gamma-glutamyl transpeptidase (GGT) by the method of Szasz [29] using the ELITech Clinical Systems Kit (France). The measured transmittance was used to calculate activity value via the following formula: Activity (U/L) = ΔA/min × 1746.

### Iron profile

Levels of serum iron and total iron binding capacity (TIBC) were measured using Spinreact Kits (Catalog #TKBSIS24-I and #BSIS25-1, respectively, S.A./S.A.U. Ctra. Santa Coloma, 7 E- 17176 Sant Esteve De Bas (GI) Spain). Serum ferritin was measured with Mouse Ferritin ELISA Kit (FTL) (ab157713, Abcam). The following formula was used to calculate transferrin saturation index: saturation (%) = (Plasma iron/TIBC) × 100.

### Histopathology

Histopathological changes were examined in spleens at Day 7 after radiation exposure. Spleen tissues of the different groups were fixed in 10% neutral formalin solution, sectioned, submitted into cassettes, fixed overnight, sectioned into 4 μm-thick sections and stained with hematoxylin and eosin (H&E). Morphometric study for megakaryocytes was performed using computer-assisted digital image analysis for quantification.

### Single-cell gel electrophoresis (Comet assay)

DNA strand damage in the spleen was estimated using the alkaline single-cell gel electrophoresis (Comet assay) according to Sasaki *et al*. [30]. Comet assay samples were taken immediately after animal sacrificing at Day 7 post irradiation. DNA damage in mammalian cells can be quantified by this assay, a rapid and sensitive procedure in which cells are embedded in agarose, lysed in an alkaline buffer, subjected to an electric current and stained by ethidium bromide. The length over which DNA streams away from the nucleus is an indicator for DNA damage, as relaxed and broken DNA fragments stream further than intact DNA. Microscopic analysis (50 nuclei/slide for a group) was carried out, and the quantity of damaged DNA migrated in the tail was recorded for each nucleus as total fluorescence percentage (percent DNA in tail). The pictures were captured with an Olympus BX43 camera attached to an Olympus PEN Lite E-PL3 camera. Open Comet v1.3.1 was used for the analysis of DNA content in the head and tail.

### Statistical analysis

GraphPad Prism® software version 7 was used for statistical analysis. Values are reported as mean ± standard error of the mean, and one-way analysis of variance coupled with the Newman–Keuls multiple comparison test was used to determine the significance of differences between mean values. Significance was determined at *P* < 0.05. For the figures, ^*^, ^*^^*^, ^*^^*^^*^ and ^*^^*^^*^^*^ indicate statistical significances of *P* < 0.05, 0.01, 0.001 and 0.0001, respectively.

## RESULTS

### MRN-100 maintained normal body weight

**Fig. 1:**
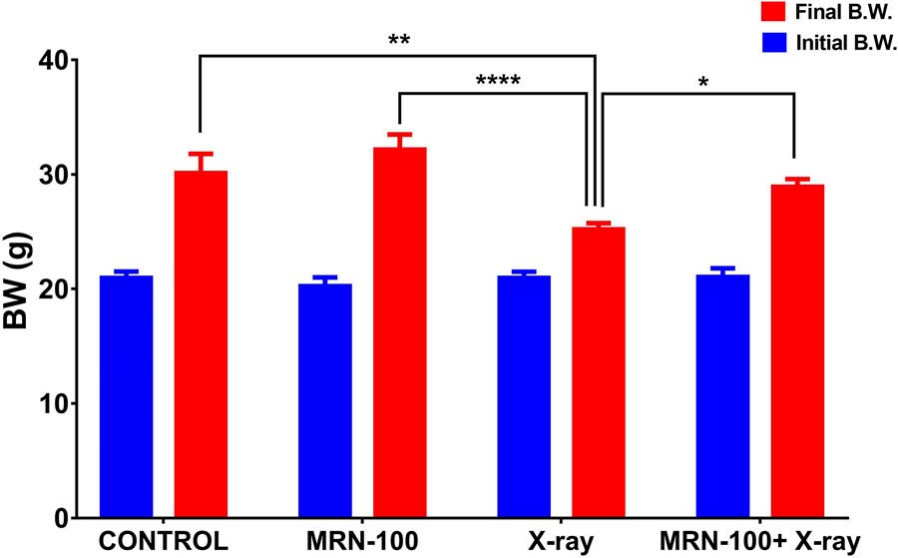
Effect of MRN-100 on initial and final BW of irradiated mice at Day 7 post irradiation; each value represents the mean ± SEM for 8, 8, 12 and 12 mice for control, MRN-100, X-ray and MRN-100 + X-ray groups, respectively, at Day 0 and Day 7 post irradiation; ^*^, ^*^^*^ and ^*^^*^^*^^*^ indicate statistical significance between groups at *P* < 0.05, *P* < 0.01 and *P* < 0.0001, respectively.

BW changes over the experimental duration are shown in Fig. 1 for mice under different treatment conditions. Mice without irradiation (MRN-100-only and control) had similar weight gains over the experiment’s duration. Irradiated mice lost BW, with a BW change of 15.3% (*P* < 0.01) compared to the control at Day 7 post-irradiation. Treatment with MRN-100 significantly limited this BW loss to be only 3.95% relative to the control group.

### MRN-100 protected spleen weight

**Fig. 2:**
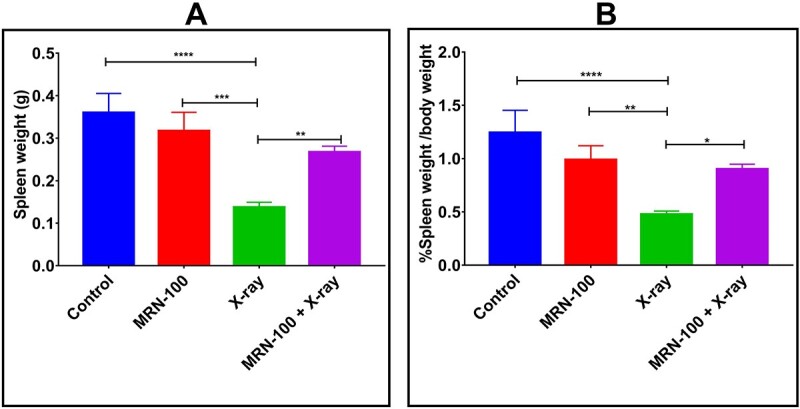
(**A**) Effect of MRN-100 on spleen weight of irradiated mice at Day 7 post irradiation; (**B**) effect of MRN-100 on relative spleen weight of mice exposed to irradiation; each value represents the mean ± SEM for 8, 8, 12 and 12 mice for control, MRN-100, X-ray and MRN-100 + X-ray groups, respectively, at Day 7 post irradiation; ^*^, ^*^^*^, ^*^^*^^*^ and ^*^^*^^*^^*^ indicate statistical significance between groups at *P* < 0.05, *P* < 0.01, *P* < 0.001 and *P* < 0.0001, respectively.

Spleen weight and relative spleen weight changes in irradiated mice at Day 7 are shown in Figs. 2A and B. Exposure to radiation caused significant spleen weight loss of 62.8% (*P* < 0.0001) and relative spleen weight loss by 61.7% (*P* < 0.0001). In contrast, MRN-100 treatment prevented irradiated mice from losing spleen weight, as it was insignificantly different from the control and MRN-100 groups.

### MRN-100 treatment protected complete blood count parameters

**Fig. 3:**
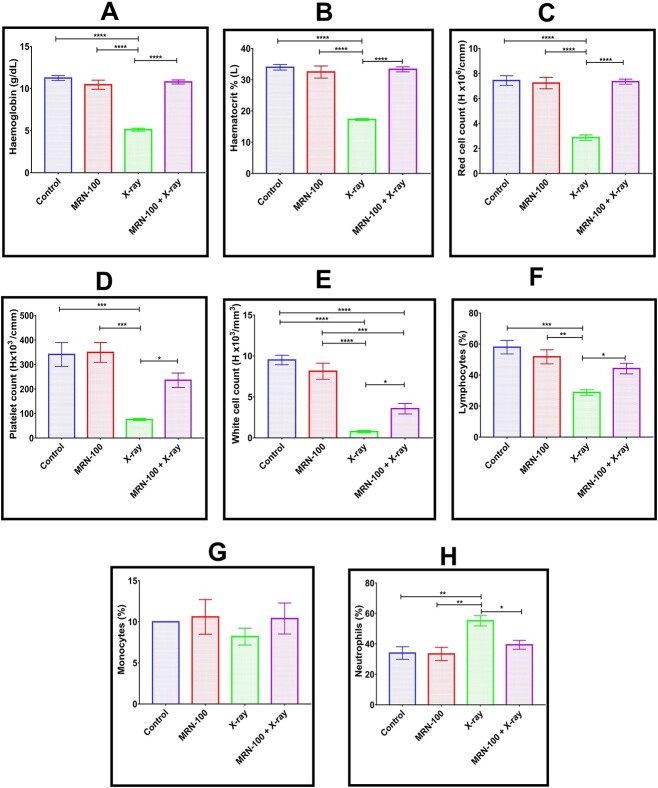
Effect of MRN-100 treatment on CBC parameters of irradiated mice at Day 7 post irradiation; (**A**) Heamoglobin (g/dl); (**B**) Hct % (l); (**C**) RBCs (10^6^ cells/cmm); (**D**) Platelets (10^3^/cmm); (**E**) WBCs (10^3^/cmm); (**F**) Lymphocytes (%); (**G**) Monocyte (%); (**H**) Neutrophils (%); each value represents the mean ± SEM for five mice for each group, at Day 7 post irradiation; ^*^, ^*^^*^, ^*^^*^^*^ and ^*^^*^^*^^*^ indicate statistical significance between groups at *P* < 0.05, *P* < 0.01, *P* < 0.001 and *P* < 0.0001, respectively.

Radiation exposure markedly decreased complete blood count (CBC) parameters, including Hb, HCT, RBC’s and platelets by 54.5%, 49.2%, 61.4% (*P* < 0.0001) and 77.8%, (*P* < 0.001), respectively, when compared to normal animals (Fig. 3). It also caused a significant decrease in WBCs (91.94%, *P* < 0.0001), lymphocytes (50.3%, *P* < 0.001) and monocytes (18%) and an increase in neutrophils by 62.4% (*P* < 0.01) versus untreated control. In contrast, MRN-100 supplementation to irradiated mice ameliorated the levels of the aforementioned parameters to nearly reach the normal values.

### Spleen histopathology

**Fig. 4:**
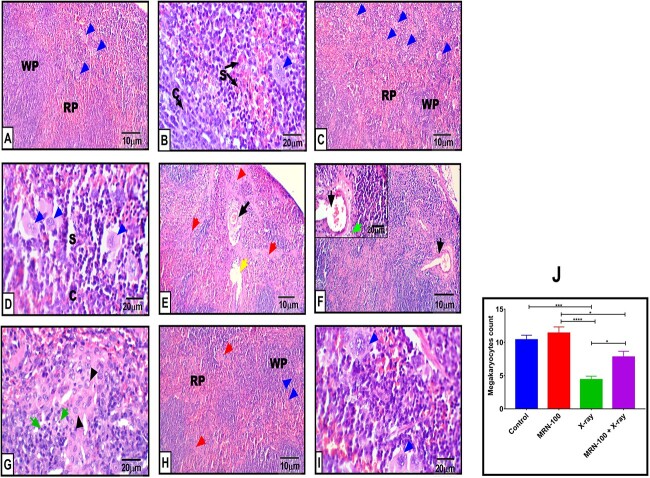
Representative H&E sections of splenectomy specimens of different groups at Day 7 post irradiation; (**A**) Control untreated group depicts a normal splenic parenchyma with the white pulp follicular zone (WP) enclosing the red pulp (RP) compartments; note the megakaryocyte (blue arrow) (100×); (**B**) higher magnification of control splenic red pulp region shows normal splenic sinuses (S) filled with peripheral blood elements such as RBCs, neutrophils, monocytes and lymphocytes penetrating the splenic cords (**C**) and a megakaryocyte (blue arrow) (400×); (C) MRN-100-treated mice display normal splenic architecture with slightly expanded red pulp (RP) rather than white pulp (WP); note the number of megakaryocytes (blue arrows) (100×); (**D**) higher magnification of MRN-100-treated group shows longitudinally arranged sinuses (S) passing through the splenic cords (C) and megakaryocyte (blue arrow) (400×); (**E**) irradiated animals reveal multiple necrotic foci (red arrow), angiectatic arteriole associated with an area of fibrosis or scarring (black arrow) and focal degeneration (yellow arrow) (100×); (**F**) splenic tissue from irradiated group shows another focus of angiectatic arteriole (black arrow) in low power (100×); insertion showing its discontinued endothelium, hyperchromatic nuclear pleomorphism around and numerous apoptotic figures in the lower level (green arrow) (400×); (**G**) splenic tissue from irradiated group depicts the atypical cellular infiltrate consisting of large atypical lymphoid cells with vesicular chromatin and small visible nucleoli (head arrow) with increased apoptosis in the background (green arrow) (400×); (**H** & **I**) splenic parenchyma from MRN-100 + X-ray treated animals reveals protected red pulp (RP), white pulp (WP) and megakaryocytes (blue arrow) in low and high power (100 and 400×, respectively); (**J**) the quantification of megakaryocytes of the different groups (I); values are expressed as the means ±SEM. ^*^, ^*^^*^^*^ and ^*^^*^^*^^*^ indicate statistical significance between groups at *P* < 0.05, *P* < 0.001 and *P* < 0.0001, respectively.

Figure 4 shows representative photomicrographs from the histopathological examination of mice under different treatments. Examination of splenic parenchyma excised from untreated control revealed normal distribution of the white pulp with its dominant B and T lymphocytes characterized by condensed chromatin and moderate amounts of eosinophilic cytoplasm, bordering the red pulp’s cords, sinuses and megakaryocytes. MRN-100 treatment showed normal splenic architecture with marked expansion of red pulp zone and an increased number of megakaryocytes (9.6%), in agreement with the biochemical investigations. On the other hand, necrotic foci were clearly observed in the splenic parenchyma after exposing to X-ray irradiation. The last is accompanied with atypical lymphoid infiltration of both centric and acentric nuclear contour, fibrosis and angiectatic arterioles. A significant decrease in the megakaryocyte number was observed (57.7%, *P* < 0.001) compared to the normal control. Pretreatment with MRN-100 markedly protected the splenic tissue from the irradiation’s adverse side effects and revealed a quite normal parenchyma and megakaryocyte number (77.3%, *P* < 0.05) with respect to the untreated irradiated group, though still suffering from some necrotic foci.

### MRN-100 ameliorated oxidative stress and antioxidant biomarkers

**Fig. 5:**
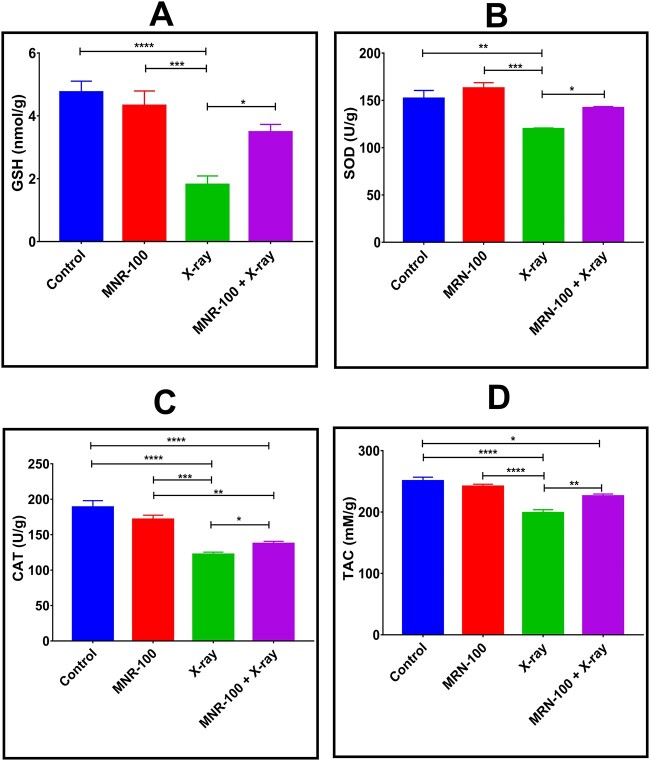
Effect of MRN-100 on the level of antioxidant biomarkers at Day 7 post irradiation: (**A**) GSH, (**B**) SOD, (**C**) CAT and (**D**) TAC in the spleen of mice exposed to irradiation; each value represents the mean ± SEM for five animals per group. ^*^, ^*^^*^, ^*^^*^^*^ and ^*^^*^^*^^*^ indicate statistical significance between groups at *P* < 0.05, *P* < 0.01, *P* < 0.001 and *P* < 0.0001, respectively.

Data in Fig. 5 show that mice treated with X-ray irradiation had significantly decreased levels of GSH, SOD, CAT and TAC by 61.9% (*P* < 0.0001), 21.2% (*P* < 0.01), 35.34% (*P* < 0.0001) and 20.70% (*P* < 0.0001), respectively, in comparison with control. This decrease in GSH content, SOD levels and CAT levels was markedly enhanced in the group exposed to irradiation and treated with MRN-100, reaching normal values.

**Fig. 6:**
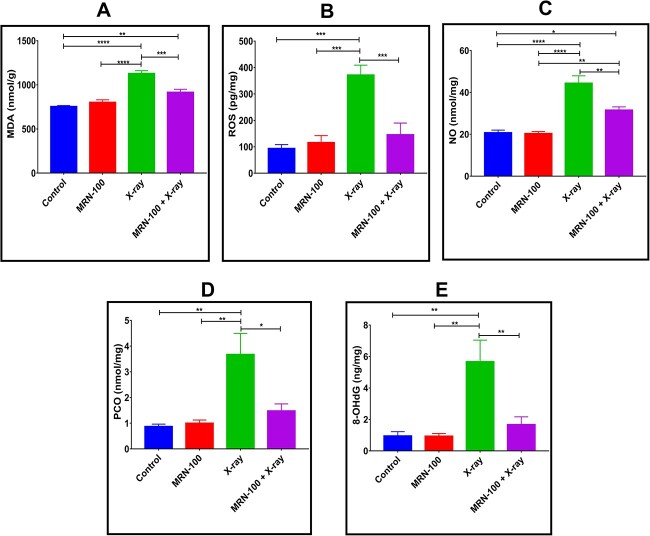
Effect of MRN-100 on the level of oxidative stress biomarkers at Day 7 post irradiation: (**A**) MDA, (**B**) ROS, (**C**) NO, (**D**) PCO and (**E**) 8-OHdG in the spleen of mice exposed to irradiation; each value represents the mean ± SEM for five animals per group; ^*^, ^*^^*^, ^*^^*^^*^ and ^*^^*^^*^^*^ indicate statistical significance between groups at *P* < 0.05, *P* < 0.01, *P* < 0.001 and *P* < 0.0001, respectively.

The radioprotective effect of MRN-100 was also noticed by its effect on LPx as measured via spleen MDA levels. Figure 6 shows MDA levels for different groups. No significant difference was observed between the control and MRN-100-only groups. Mice exposed to irradiation had markedly elevated MDA levels by 49.70% (*P* < 0.001) relative to the untreated control. Mice treated with MRN-100 before irradiation had a significant reduction in MDA by 21.48% as compared to the normal values (Fig. 6A). Similarly, ROS levels were significantly increased by 297.7% (*P* < 0.001) in spleen tissues of irradiated animals relative to the control, but this elevation was markedly reduced in the co-treatment group where it reached only 55.8% of control (Fig. 6B). In addition, Figs 6C and D also shows that NO and PCO levels were markedly elevated in the spleen homogenate of irradiated mice relative to the untreated controls by 113.74% and 323.47% (*P* < 0.001), respectively. In contrast, supplementation of MRN-100 to irradiated animals significantly suppressed the levels of NO and PCO to reach 51.88% and 70.59% of the control values, respectively. Finally, 8-OHdG level was highly increased in splenic tissue by 494.96% (*P* < 0.01) of control post exposure to irradiation, while MRN-100 intake by mice prior to irradiation effectively reduced such elevation to reach only 74.88% as compared to the untreated controls.

### Comet assay

**Fig. 7:**
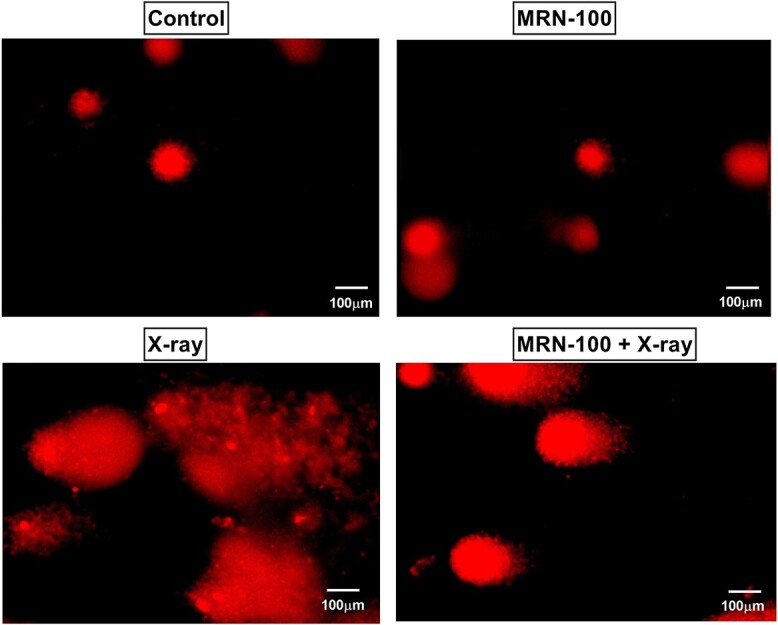
Representative photomicrographs that demonstrate DNA damage in spleen tissues using comet assay at Day 7 post irradiation for the control group, MRN-100 group, X-ray group and MRN-100 + X-ray group.

DNA damage in spleen cells was measured using single-cell gel electrophoresis and measuring tail moment, length and DNA percentage (Fig. 7 and Table 1). The spleen cells of mice exposed to X-rays showed a highly significant increase in DNA fragmentation as represented by the measured tail moment, length and DNA percentage relative to the control group. Such damage represents increases by 1835%, 184% and 613%, respectively, over the control group values. Conversely, spleen cells of mice pretreated with MRN-100 showed a significant reduction in the various comet parameters induced by X-ray exposure, suggesting there is enhanced repair resulting from MRN-100 administration to X-ray treated mice. A slight increase in tail moment, length and DNA percentage by only 500%, 61% and 280%, respectively, versus control group was recorded.

**Table 1:** Comet assay parameters obtained by image analysis in spleen cells

**Groups**	**Control**	**MRN-100**	**X-ray**	**MRN-100 + X-ray**
% DNA in tail	2.62 ± 0.43	3.78 ± 0.45	18.77 ± 0.90^a,b^	9.10 ± 0.70^a,b,c^
Tail length (μm)	1.10 ± 0.09	1.29 ± 0.13	3.13 ± 0.16^a,b^	1.78 ± 0.19^a,c^
Tail moment (Unit)	2.93 ± 0.52	4.41 ± 0.61	56.7 ± 3.34^a,b^	17.58 ± 2.68^b,c^

Each value represents the mean ± SE of five mice in each group.

^a^Significantly different from the control group at 0.01 and 0.05 level, respectively. ^b^Significantly different from the MRN-100 group at 0.01 and 0.05 level, respectively. ^c^Significantly different from the X-ray group at 0.01 level.

### Iron profile

**Fig. 8:**
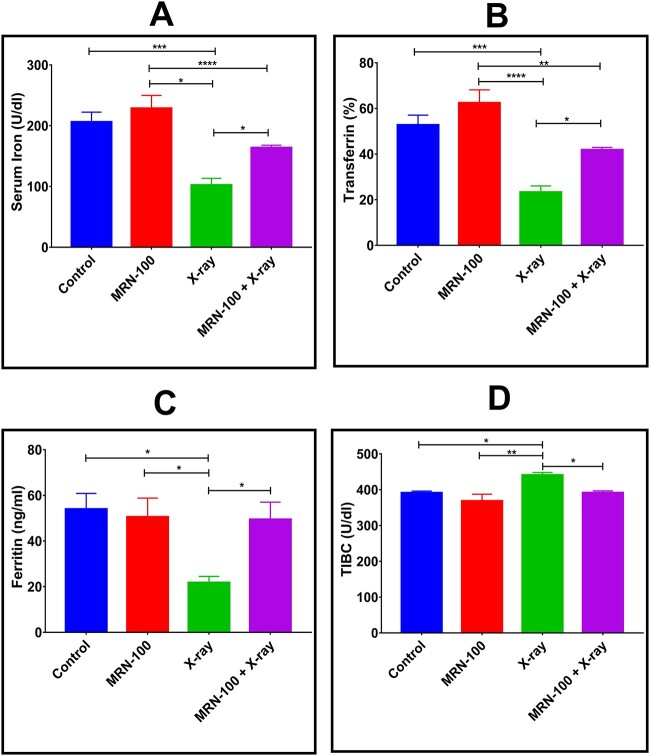
Effect of MRN-100 administration on serum levels of (**A**) total iron, (**B**) transferrin, (**C**) ferritin and (**D**) TIBC at Day 7 post irradiation; data are means of five mice/group ± SEM; ^*^, ^*^^*^, ^*^^*^^*^ and ^*^^*^^*^^*^ indicate statistical significance between groups at *P* < 0.05, *P* < 0.01, *P* < 0.001 and *P* < 0.0001, respectively.

As shown in Fig. 8, mice exposed to X-rays showed serum iron, transferrin and ferritin concentrations that were significantly reduced by 50.3% (*P* < 0.001), 55.9% (*P* < 0.001) and 59.7% (*P* < 0.05), respectively, whereas serum TIBC was significantly elevated by 12.6% (*P* < 0.05) with respect to normal control. However, MRN-100 supplementation to irradiated mice significantly (*P* < 0.05) increased serum values of iron, ferritin and transferrin value, and decreased (*P* < 0.05) serum TIBC with respect to untreated irradiated mice.

### MRN-100 modulated liver function enzymes

Liver enzyme levels of ALT, AST and GGT in the serum are shown in Table 2. MRN-100 administration to mice revealed insignificant changes in comparison to the untreated control. Irradiated animals had significantly elevated levels of liver enzymes by 96.09%, 342.39% and 117.24% for ALT, AST and GGT, respectively, relative to the untreated control. Meanwhile mice in the MRN-100 + X-ray group had significantly decreased liver enzyme levels: 34.75% for ALT, 78.17% for AST and 88.07% for GGT relative to the untreated control.

**Table 2:** Effect of MRN-100 and/or irradiation treatment on liver function enzymes

**Groups**	**Control**	**MRN-100**	**X-ray**	**MRN-100 + X-ray**
ALT (U/L)	36.32 ± 3.113	39.32 ± 3.122	71.22 ± 2.604^a,b^	48.94 ± 2.774^c^
% change from control		8.26%	96.09%	34.75%
AST (U/L)	39.4 ± 0.822	40.56 ± 2.358	174.3 ± 32.08^a.b^	70.2 ± 15.74^c^
% change from control		2.94%	342.39%	78.17%
GGT (U/L)	24.48 ± 6.179	22.86 ± 5.721	53.18 ± 9.227^a,b^	46.04 ± 2.667
% change from control		−6.62%	117.24%	88.07%

Each value represents the mean ± SEM of five samples/group.

^a^Significantly different from the Control group at the 0.01 and 0.05 levels, respectively. ^b^Significantly different from the MRN-100 group at the 0.01 and 0.05 levels, respectively. ^c^Significantly different from the X-ray group at the 0.01 level.

## DISCUSSION

Although radiation therapy is the mainstay treatment for cancers, excessive exposure to IR can cause severe damage to many organs in the body, including those of the hematopoietic tissue [31–33], mandating the development of protective nutraceuticals. MRN-100 is a hydro-ferrate fluid, an iron-based compound composed of bi- and trivalent ferrates isolated from phytosin. We have earlier shown that MRN-100 is a potent antioxidant as evidenced by its ability to protect against *γ*-radiation-induced hematopoietic tissue damage and lethality in the fish Nile tilapia, *Oreochromis niloticus*, *in vivo* [14]. Treatment with MRN-100 enhanced the *γ*-radiated fish survival rate by 3.2-fold and provided protection for the RBC series, the total WBC count and the histopathology of various organs of irradiated fish [14]. Therefore, we carried out the current study to examine this potential effect in mice.

Results showed that MRN-100 supplementation protects mice against oxidative stress caused by X-ray irradiation. This protective effect is linked with MRN-100’s ability to alleviate the oxidative stress in rats and *in vitro* [15, 16], suggesting that MRN-100 could potentially be used as a radioprotector. MRN-100 is well known for its established health benefits. It promotes an anti-inflammatory response *in vitro* [17] and has shown to have potential as a protector against the following: oxidative stress *in vivo* and *in vitro* [15, 16], *γ*–radiation [14], HIV activity [18] and chemically induced esophageal and gastric cancer in rats [19].

Studies with IR have shown that the most radiosensitive tissues are hematopoietic tissues. Our current study also found a significantly depressed CBC of X-ray-treated mice, and spleen histopathology showed necrotic foci, fibrosis and angiectatic arterioles in the splenic parenchyma after exposing to X-ray irradiation. These results are in accordance with others [5–7, 33]. Furthermore, rats exposed to gamma radiation have shown histomorphological alterations of the spleen [34] and mice exposed to X-rays have shown a decrease of splenic lymphocytes [35]. In contrast, the administration of MRN-100 results in significant safeguarding against radiation-induced harm to various CBC metrics, including Hb, HCT, RBC’s, platelets, WBC count, lymphocytes and monocytes. Moreover, histopathology of spleens from the MRN-100 + X-ray-treated mice reveals protected red pulp, white pulp and megakaryocytes, as compared with X-ray exposure alone that showed multiple necrotic foci, fibrosis, focal degeneration and numerous apoptotic figures. Other radioprotectors like pegylated G-CSF have been found to improve mice survival after whole-body ionizing irradiation exposure, inhibit depletion of WBC and platelets and sustain RBC balance and hemoglobin levels after exposure to radiation [36]; and aloe vera alters the X-ray-induced damage that mice sustain in splenic and hematological tissues [37].

MRN-100’s ability to prevent oxidative stress damage to tissues could involve the regulation of free iron levels in cells. MRN-100 intake enhanced plasma iron, ferritin concentrations and transferrin saturation, which are known to be important for protection against oxidative stress [38, 39]. These results agree with our prior studies showing enhancement of ferritin concentrations, plasma iron and transferrin saturation of aged mice post exposure to MRN-100. Those results showed that MRN-100 can reverse oxidative changes related to aging and even shift levels so that they are within the same level ranges of young control rats for various tissues such as the brain, liver and blood [16].

Here, MRN-100 exerts a significant radioprotective effect, but the mechanisms underlying its effect are not fully known. MRN-100 could potentially prevent reactive radical accumulation by preventing excess iron from participating in the Fenton reaction. The ROS reduction due to MRN-100 may be a mechanism by which it exerts radioprotective effects. High concentration of ROS influences the evolution of many diseases, including cancer [40] and various neurodegenerative diseases [41]. Results of the present study demonstrate MRN-100’s antioxidative activity in X-ray-treated mice. Spleen tissue measurements focusing on LPx show that MRN-100 significantly protects against radiation-induced MDA elevation and inhibits modifications to the levels of another oxidative stress biomarker (8-OHdG) for DNA damage in cells, NO and PCO groups.

The present study also shows that MRN-100 treatment prevented a decrease in GSH, an effect that is of particular interest due to GSH’s major role in the endogenous antioxidant system that inhibits the neoplastic process. In addition, MRN-100 prevented declines in the antioxidant enzymes CAT and SOD, in agreement with MRN-100’s reported antioxidant activity in other models. MRN-100 has been shown to be a powerful antioxidant in chemically induced esophageal and gastric cancers in Wistar rats by elevating GSH and antioxidant enzymes and TAC levels that occur together with reductions in MDA and total free radical levels [19]. Another model showed that MRN-100 supplementation in rats reversed aging by inhibiting the oxidative stress biomarker levels of PCO groups, NO, MDA and total free radicals that was accompanied by an enhancement of GSH content and the antioxidant enzymes [16]. These antioxidant enzymes must be present for the clearance of hydrogen peroxide and superoxide [42]. The above-mentioned three models represent a mechanism for MRN-100’s radioprotective effects, illustrating the reduction in ROS by this agent. The results conform with those of other agents, including results showing that wheat germ oil and bone marrow transplantation to gamma-irradiated rats cause significantly decreased MDA levels and significantly elevated GSH and IL-6 levels as compared with only irradiated rats [43]. MGN-3/Biobran, an arabinoxylan, has also been shown to ameliorate the elevation of MDA and protect against irradiation-induced decline of GSH in the spleens of mice [44].

The immunomodulatory effects of MRN-100 may also be key to its antiradiation effects. Radiation’s ability to suppress the immune system is well documented in animals and humans. Epidemiological studies of IR have shown that it can impair immune responses in a dose-dependent manner, cause a persistent state of inflammation and deregulate the production of cytokines [45, 46]. Radiation exposure also significantly depresses RBCs, WBCs, lymphocytes and monocyte count and can lead to dysfunction for various immune cell populations [14, 43, 47–51]. The present study shows that mice treated with X-rays alone had a significant decrease in WBC count, lymphocyte and monocyte percentages by 91.94%, 50.3% and 18%, respectively, while neutrophils revealed a marked increase by 62.4% in comparison to the untreated control. In contrast, MRN-100 pretreatment to mice irradiated with X-rays ameliorated the percentage of lymphocytes and neutrophils, reaching similar values as the control.

Our recent study of human dendritic cells (DCs) *in vitro* also showed that MRN-100 can have an anti-inflammatory effect [17]. MRN-100 can increase the production of interleukin IL-10, an anti-inflammatory cytokine, and it can upregulate the co-stimulatory molecules CD86 and CD80 in DCs. In addition, pretreating DCs with MRN-100 enabled them to prime CD4+ T cells to secrete significant amounts of IL-10 while inhibiting the secretion of tumor necrosis factor-α, a pro-inflammatory cytokine. It is of interest to see extensive studies showing the augmented expression of IL-10 as an important mechanism for different agents that have been examined for their effects against IR. These include the attenuation of testicular dysfunction in rats by alpha-lipoic acid [52], anti-photoaging effects of low molecular-weight fucoidan [53] and protection against immune suppression in mice by N-acetyl tryptophan glucoside [54]. These results demonstrate MRN-100’s power as an anti-inflammatory agent that promotes anti-inflammatory immune responses *in vitro*.

Our earlier study also showed that MRN-100 possesses protective effects against human immunodeficiency virus (HIV) *in vitro*. HIV-1 replication was inhibited in samples (79%) by MRN-100 (of concentration 10% v/v), and MRN-100 led to improved survival for HIV-1-infected peripheral blood mononuclear cells [18]. These results suggest that MRN-100 may help to protect against irradiation-associated immune dysfunction, i.e. MRN-100 could be an immunomodulator in addition to being a radioprotector.

Our results examined MRN-100’s ability to provide protection against DNA damage as a possible mechanism of its anti-radiation effects. 8-OHdG, a marker specific to DNA damage mediated by ROS, was highly increased in splenic tissue post exposure to irradiation. However, MRN-100 intake to mice prior to irradiation effectively reduced such elevation. Our result is consistent with Gao *et al*. [55] who found an elevation in 8-OHdG level in peripheral blood indicating oxidative damage in radiation workers who were exposed through their occupation to low dose X-radiation. Assessment of DNA damage was also carried out by Comet assay. Spleen cells’ DNA damage was measured using single-cell gel electrophoresis. Though there are two types of tails in the Comet assay, the measured comet tails of this study can be attributed to DNA damage. This conclusion is supported by the sudden increase in free radicals and corresponding decline in CBC parameters, which are indicative of radiation exposure, as well as by the elevation of 8-OHdG levels, a marker specific to DNA damage. Splenic cells from mice exposed to X-rays have significantly more DNA fragmentation as represented by the measured tail moment and length relative to the control group, while splenic cells of mice pretreated with MRN-100 showed a less dramatic increase in tail moment and length. These results suggest that MRN-100 intake protected DNA from damage in spleen cells.

We chose to study male mice. In animal studies, sex was not found to be a statistically significant parameter in terms of radiation necrosis development in BALB/c and C57BL/6 mice [56]. On the other hand, a significant relative loss in cardiac contractility was found for male C57L/J wild-type mice at Days 90 and 180 post-irradiation compared to female mice [57]. Human studies have shown conflicting results as well. Earlier studies have shown that radiation effects are sex-specific and reported long-term radiosensitivity in women is higher than that in men who receive a comparable dose of radiation. In addition, women may be at significantly greater risk of suffering and dying from radiation-induced cancer than men exposed to the same dose of radiation [58]. On the other hand, recent studies have also reported no evidence for a difference in radiosensitivity between the sexes [59]. We note here that focusing on male mice is a potential limitation of the current study and suggest that future studies use both sexes.

For many radioprotectors, safety is a significant concern. Some synthetic compounds exert radioprotective effects but are known to be toxic [60, 61]. In the current study, MRN-100 pretreatment of irradiated mice provided significant protection against elevated liver enzymes ALT, AST and GGT at 1 week after irradiation. Furthermore MRN-100 is currently sold as a soft drink in Japan. MRN-100 is well known for its established several health benefits. In the current study, we show MRN-100’s ability to act as a powerful radioprotector using a mice model, and we further clarify the possible mechanisms underlying its radioprotective effect. Our results suggest that MRN-100 could be useful as an adjuvant treatment that counteracts the severe damage caused to vital organs by radiation exposure.

## CONCLUSION

In conclusion, MRN-100 intake was demonstrated to protect mice against the harmful effects of IR. MRN-100 has the potential to protect against oxidative stress caused by radiation exposure, suggesting its potentially beneficial use as an adjuvant treatment that counteracts radiation therapy’s severe adverse side effects.

## Data Availability

The data that support the findings of this study are available from the authors upon reasonable request from the corresponding author.
